# Assessment of Insulin Stability Inside Diblock Copolymer PEG-PLA Microspheres

**DOI:** 10.3797/scipharm.1002-01

**Published:** 2010-06-23

**Authors:** Mohamed Abbas Ibrahim

**Affiliations:** Kayyali Chair for Pharmaceutical Industries, Department of Pharmaceutics, Faculty of Pharmacy, King Saud University, Riyadh, Saudi Arabia

**Keywords:** Bovine insulin, Diblock, Copolymers, Microspheres, Stability

## Abstract

Insulin-loaded PEG2-PLA40 and PEG5-PLA20 microspheres containing 5% bovine insulin were manufactured using single emulsion and w/o/w multiple emulsion-solvent evaporation techniques. Microspheres were characterized for their insulin encapsulation efficiency and release characteristics in phosphate-buffered saline (PBS) at pH 7.4 and 37 °C. Moreover, the stability of the peptide during 18 days of release was evaluated using HPLC and HPLC-MS techniques. The results showed that the loading efficiencies were higher in case of insulin loaded PEG2-PLA40 and PEG5-PLA20 microspheres prepared by single emulsion emulsion-solvent evaporation technique. Insulin release was characterized by an initial burst, which was attributed to the amount of protein located on or close to the microsphere surface. The total ion chromatogram (TIC) of insulin samples extracted after 6, 12 and 18 days of PEG2-PLA40 microspheres erosion showed that insulin was intact inside the eroding microspheres. In addition, only small amounts of protein undergo degradation under these conditions (only 11.69% ± 1.13 of the initially loaded insulin loading were detected as degradation products after 18 days. Mass spectra recorded at these retention times confirmed the presence of insulin with a molar mass of 5734 Da and other two products of molar masses of 5587 Da and 5487 Da.

## Introduction

Besides the limited stability of proteins and peptides in the biological environment, their integrity was also strongly affected by processing and storage conditions [1]. Therefore, any formulation strategy in which a protein or peptide drug is involved has to be carefully planned to retain the biological activity during processing, storage and in vivo application. In this respect, adjuvants play an important role. The search for stabilizing agents has led to extensive investigations on the potential of biodegradable polymers for the controlled delivery of protein and peptide drugs [2]. A special focus has thereby been on the exploration of the potential of poly (α-hydoxy acids) such as poly(lactic acid) (PLA) and poly(lactic-co-glycolic acid) (PLGA), all of which had originally been approved as degradable suture materials [3]. Despite the success of this strategy, PLA and PLGA may not provide a microenvironment that is unequivocally suitable for all protein and peptide drugs [4]. Since most representatives of this class of drugs are subject to physical and chemical instability, it turned out to be a major challenge to design controlled release devices that preserve the biological activity of proteins and peptides [5]. Consequently, major research efforts have been undertaken to investigate the impact of processing techniques as well as to reveal the physicochemical conditions that prevail during PLA and PLGA erosion and the effect thereof on the stability of drugs [6]. Elevated levels of moisture, providing sufficient protein mobility for reactivity, the acidic microclimate induced by acidic degradation products, the carboxylic acid end groups on the polymer and protein adsorption on polymer surfaces are a few of the innumerable parameters that were identified to cause protein instabilities, mainly via unfolding and aggregation [4].

The combination of biocompatible poly(ethylene glycol) (PEG) with biodegradable PLA or PLGA by either blending the polymers [7] or by synthesizing block copolymers [8] is an approach that has been followed by a range of groups to enhance protein stability in biodegradable systems. Covalently bound PEG proved to be helpful for reducing peptide and protein adsorption by masking hydrophobic polymer surfaces [9, 10] and PEG blends were successfully used to prevent microclimate-induced instability reactions of proteins in degrading polymers, probably by preventing the development of an acidic microclimate [7].

Among therapeutically active peptide molecules, insulin is of great interest for its wide use in the treatment of diabetes mellitus. Bovine insulin consists of 51 amino acids in two chains, a 21-residue A-chain and a 30-residue B-chain, linked by two disulfide bonds [11]. The chain A contains additional disulfide loop between A6 and A11. In our previous study [12], insulin-loaded PLA and PLGA microspheres were characterized for their encapsulation efficiencies, in vitro protein release and integrity during microspheres erosion. The results revealed that deamidation was the major mechanism of instability. Surprisingly, no acylation products were found. Control experiments in concentrated lactic acid solutions confirmed a minimal reactivity of the peptide under these conditions.

In this paper, the microencapsulation of bovine insulin using biodegradable diblock copolymers, PEG2-PLA40 and PEG5-PLA20 is investigated using single w/o emulsion and w/o/w multiple emulsion-solvent evaporation techniques. The effects of diblock copolymers on the in-vitro release properties of the protein microspheres, as well as protein stability inside the eroding microspheres are studied.

## Results and Discussion

**Fig. 1:**
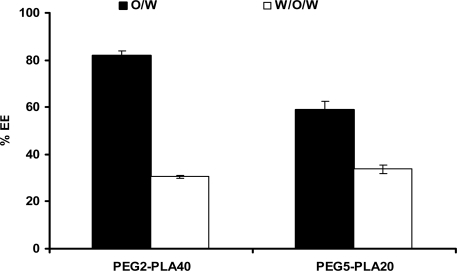
Effect of the microencapsulation process on the entrapment efficiency percentage (% EE) of insulin-loaded biodegradable diblock copolymers.

The entrapment efficiencies of the protein inside PEG-PLA microspheres prepared by multiple emulsion solvent evaporation technique were found lower than that obtained using single emulsion solvent evaporation technique. The numerical values of entrapment efficiencies were displayed in figure 1. The entrapment efficiencies values were 81.9 and 58.95 for PEG2-PLA 40 and PEG5-PLA 20 microspheres, respectively, prepared by single emulsion method, while 30.47 and 33.72 % entrapment efficiencies were obtained with PEG2-PLA 40 and PEG5-PLA 20 microspheres, respectively, prepared by multiple emulsion method.

The lower entrapment efficiency of insulin in case of PEG5-PLA20 microspheres prepared by single emulsion is lower than PEG2-PLA40 microspheres. The higher PEG content of PEG5-PLA20 molecule might be the reason for such reduction in the entrapment efficiency. Covalently bound PEG proved to be helpful for reducing peptide and protein adsorption by masking hydrophobic polymer surfaces. Therefore, the PEG-PLA polymer that carries more hydrophilic PEG moiety is expected to escape from the organic ethylacetate phase during microspheres preparation, resulting in lower entrapment efficiency.

### Microspheres morphology

**Fig. 2:**
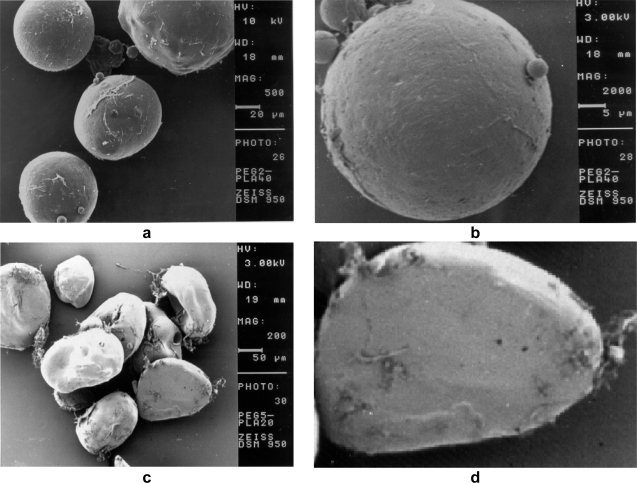
Scanning electron microscopy (SEM) images of PEG5PLA20 and PEG2PLA40 microspheres containing bovine insulin as obtained after manufacturing and freeze-drying. (a) PEG2PLA40 particles at low magnification. (b) PEG5PLA20 particles at low magnification. (c) PEG2PLA40 particles at high magnification. (d) PEG5PLA20 particles at high magnification.

The manufactured PEG2-PLA40 and PEG5-PLA20 microspheres prepared using the single emulsion-solvent evaporation technique were characterized for their surface morphology using the scanning electron microscopy (SEM), Fig. 2. The SEM images showed that the produced PEG2-PLA40 microspheres were clearly spherical with slightly porous and rough surfaces surface. Uchida et al. [13] attributed the microsphere surface pores to organic solvent that diffused from the particle core to the surface during the particle hardening. However, PEG5-PLA20 microspheres were found to be of irregular morphology but with smooth surfaces. In addition, the images reveal that insulin crystals were found in the image field, which might be responsible for the higher initial release from such microspheres.

### In vitro release from PEG-PLA microspheres

**Fig. 3:**
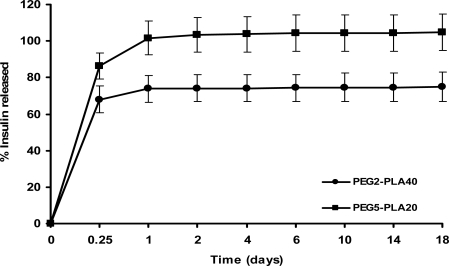
Release profile of insulin from loaded PLA (white circles) and PLGA (black squares) microspheres (mean values ± S.D.).

The in vitro release investigations from the manufactured peptide-loaded microparticles were conducted so as to ensure that insulin was entrapped in the microspheres over a sufficiently long time interval for the protein to interact with the polymer. The in vitro release profiles of insulin from PEG2-PLA40 and PEG5-PLA20 containing 5% w/w of the protein are showed in Fig. 3. The release profile of insulin from its-loaded biodegradable polymer microspheres is biphasic. A substantial initial burst release of 68.01% and 86.46% of the encapsulated protein took place from PEG2-PLA40 and PEG5-PLA20, microspheres, respectively, after 6 hr. This initial burst was followed by a very slow release along the 18 days. In addition, all entrapped insulin was released from PEG5-PLA20 microspheres during the first day. The phenomenon of initial burst of the protein from biodegradable polymer microspheres has been explained by the accumulation of the protein at the aqueous/organic interface during the solvent evaporation process and consequently, near to the surface of the microspheres [14–16]. In addition, many authors report protein retention to be a frequently encountered problem for biodegradable microspheres caused by aggregation and hydrophobic polymer–protein interactions [17, 18]. A sufficiently high amount of the protein was only retained inside PEG2-PLA40 microspheres after the release period and allowed for investigation into the stability of insulin over a period of several days. Therefore, the further studies will focus only the stability of insulin inside the eroding PEG2-PLA40 microspheres, because all the encapsulated insulin was released from PEG5-PLA20 microspheres during the first day.

### Insulin integrity inside PEG2-PLA40 microspheres during erosion

**Fig. 4:**
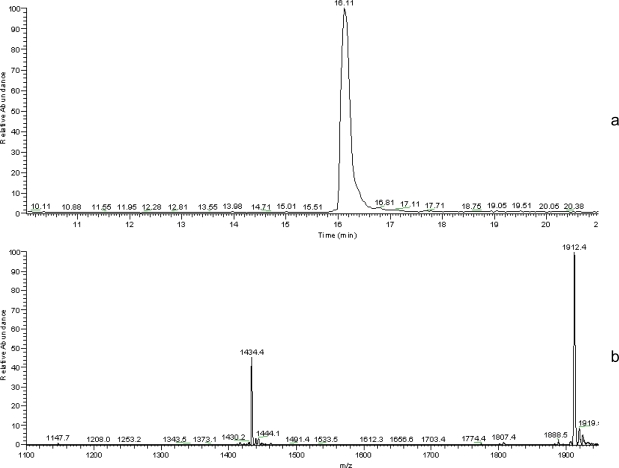
(a) HPLC-MS total ion chromatogram and (b) electrospray mass spectrum (retention time 16.11 min) of native insulin.

**Fig. 5:**
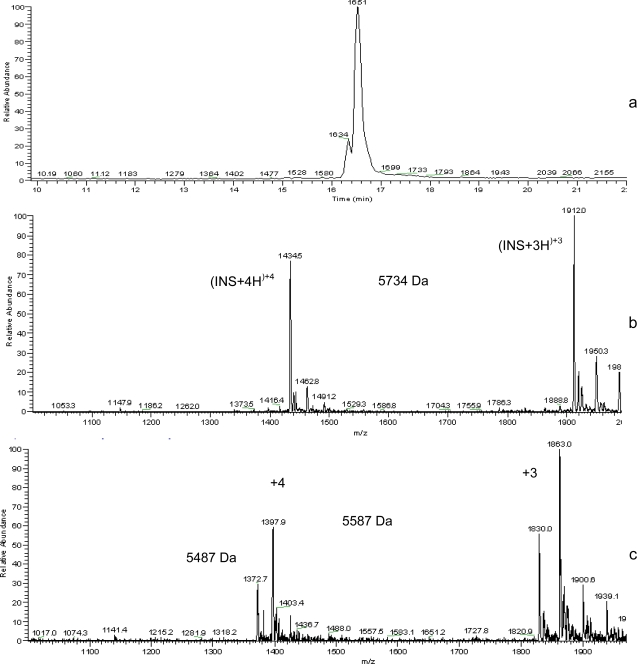
(a) HPLC-MS total ion chromatogram and (b) electrospray mass spectrum (retention time 16.516 min) of insulin; extracted from PEG2-PLA40 microspheres after 18 days release and (c) electrospray mass spectrum of degradation products (retention time 16.34 min).

After 18 days release, insulin was extracted from PEG2-PLA40 microspheres and was examined for its integrity using HPLC-MS analysis. While prior to erosion insulin was found unaltered, Fig. 4a shows the total ion chromatogram (TIC) of native insulin in which a characteristic peak appeared at a retention time of 16.63 minutes, the mass of which is 5734 Da (Fig. 4b). The total ion chromatograms (TICs) recorded after erosion for 18 days revealed again the presence of native insulin as well as a very small peak of the degradation product at retention time of 16.51 and 16.34 respectively (Fig. 5a). Mass spectra recorded at these retention times confirmed the presence of insulin with a molar mass of 5734 Da (Fig 5b) and other two products of molar masses of 5587 Da and 5487 Da (Fig. 5c).

**Fig. 6:**
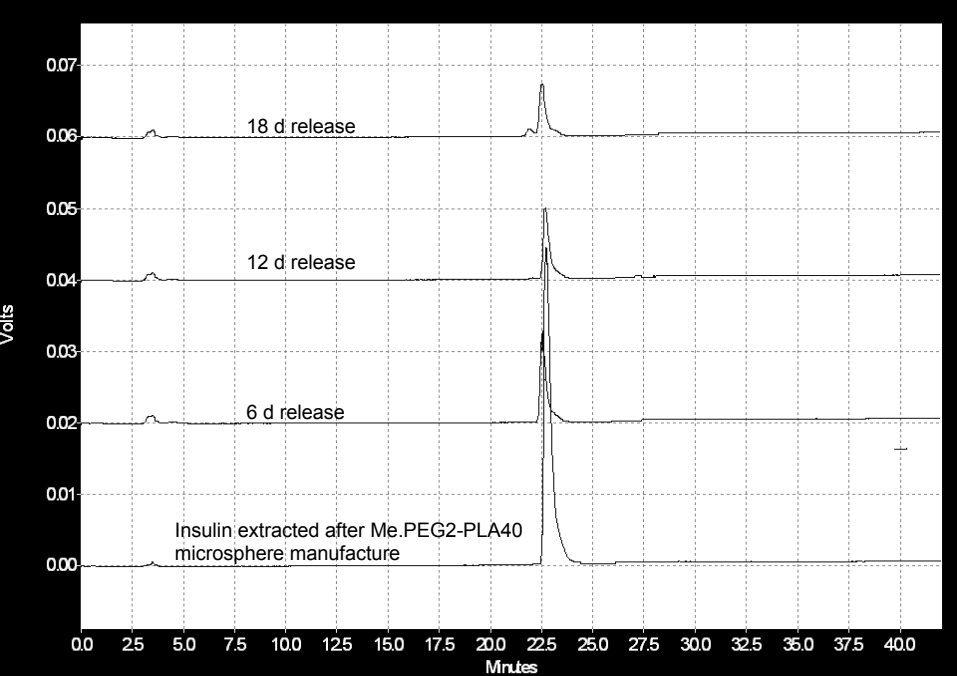
HPLC chromatograms of insulin extracted from PEG2-PLA40 microspheres after the incubation in PBS; pH 7.4 at 37 °C.

To monitor the rate of insulin degradation inside its loaded PEG2-PLA40 microspheres, the microspheres were allowed to erode under gentle shaking for 18 days. In Fig. 6, the HPLC chromatograms show the effect of incubation time on the rate of insulin degradation inside its loaded microspheres. The characteristic peak of the native insulin appeared at a retention time of 23 min. In addition, another peak begins to appear after 18 days incubation at a retention time of 21.5 min. This peak when analyzed by HPLC–MS, could be attributed to the degradation products of the protein of molar masses of 5587 Da and 5487 Da. The abundance of the degradation products could not be detected during the incubation period of 12 days, and it begins to slightly appear after 18 days of incubation.

In the present study, neither deamidation nor acylation products were detected in the HPLC-MS spectra for insulin inside the eroding PEG-PLA microspheres. However, products of the protein of molar masses of 5587 Da and 5487 Da were found in the mass spectra of insulin extracted from PEG-PLA microspheres after 18 days release. These masses may be due to the formation of insulin oligomers. The mass of 5587 Da is due to the loss of a phenylalanine peripheral residue from chain B and the mass of 5487 Da is due to the loss of a phenylalanine and valine peripheral residues from chain B. This finding was supported by Brange [19].

**Fig. 7:**
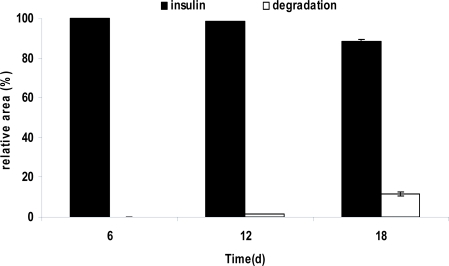
Relative peak area of insulin and its degradation products obtained from HPLC analysis data after 18 days release from PEG2-PLA40 microspheres (mean values ±S.D.).

In order to follow the rate of the insulin degradation product formation, microspheres were extracted at various time points up to 18 days of degradation. The relative area of peaks that stem from insulin and insulin degradation extracted from microsphere samples after 6, 12 and 18 days are displayed in Fig. 7. It is obvious that only small amounts of protein undergo degradation under these conditions (only 11.69% ±1.13 of the initial PEG2-PLA40 microspheres insulin loading were detected as degradation products after 18 days).

According to various literatures, microspheres made of a physical blend of PEG with PLA or PLGA had shown a protective effect on protein stability by preventing the development of an acidic microclimate [7] and by suppressing adsorption phenomena [20]. In earlier studies with Me.PEG-PLA diblock copolymers, PLA surfaces had been masked by covalently bound PEG chains, thus reducing peptide and protein adsorption [9, 21]. The copolymer poly-D,L-lactide-co-poly(ethylene glycol) (PLA-PEG) was tested for glucose oxidase entrapment. The highest protein activity was observed when the copolymer contained 10–30% PEG, which was a far better performance compared to that resulting from microspheres made from PLA or PLGA [22]. Lucke et al. [23] investigated whether the block copolymerization of PLA with PEG reduces human atrial natriuretic peptide (ANP) acylation inside degrading microspheres. When comparing these data to those obtained from degrading PLA microspheres, they showed that the relative amount of ANP-lactic acid (acylation product) inside Me.PEG5-PLA45 microspheres was only slightly but significantly (*P*, 0.05) reduced after 21and 28 days of polymer degradation.

As mentioned previously, the impact of PEG moiety in enhancing insulin stability during biodegradable microspheres erosion might be due to a fact that the covalently bound PEG proved to be helpful for reducing peptide and protein adsorption by masking hydrophobic polymer surfaces [9, 10] and probably by preventing the development of an acidic microclimate [7].

## Conclusion

The present work concludes that the encapsulation of insulin in the PEG-PLA matrices could be a protective mean in order to keep the integrity of the protein inside the eroding microspheres for 18 days. For the future, the study could be a starting step in formulating insulin microspheres that are capable of maintaining the structure integrity of the protein for a long period.

## Experimental

### Materials and methods

Poly(D,L-lactic acid)–poly(ethylene glycol)-mono-methyl ether; PEG2-PLA40 and PEG5-PLA20 were obtained from Boehringer Ingelheim (Ingelheim, Germany). Bovine insulin (51 amino acids, Mw 5734 Da) was kindly supplied by Aventis (Frankfurt, Germany). Ethyl acetate was obtained from Fluka Chemica (Buch, Switzerland). Poly(vinyl alcohol) PVA, 98% hydrolyzed (Av. Mw 13,000–23,000) was obtained from Aldrich Chemical Co. (Milwaukee, USA), 4,4′-Dicarboxy-2,2′-biquinoline (Bicinchoninic acid, BCA) from Sigma Chemical Co. (St. Louis, USA). Acetonitrile was obtained from Baker (Deventer, The Netherlands). Triflouroacetic acid (TFA) was obtained from Sigma-Aldrich (Seelze, Germany). All other reagents were of analytical grade or higher purity. Water used throughout the investigation was double-distilled and filtered through a cellulose nitrate filter with pores of 0.2 Am diameter (Sartorius, Göttingen, Germany).

### Microspheres preparation

Insulin-loaded Me.PEG–PLA microspheres were manufactured using a single-emulsion/solvent evaporation technique [23]. In brief: 300 mg polymer was dissolved in 2 ml ethyl acetate and 15 mg of bovine insulin were added to form a suspension. After addition of 4 ml of a PVA solution (1%, w/w) and homogenization by vortex mixing (Vortex, Scientific Industries, Bohemia, USA) for 10 s, the resulting o/w-emulsion was poured into a beaker containing 100 ml of PVA solution (0.5%, w/w). The mixture was stirred for 2 h at 500 rpm (Multipoint HP, Labortechnik, München, Germany) to harden the microspheres by solvent evaporation. The resulting microspheres were then collected by centrifugation and washed twice with 25 ml of water and then they were frozen in liquid nitrogen and freeze–dried.

The second method applied for the manufacture of insulin-loaded Me.PEG–PLA Microspheres is the double emulsion solvent evaporation technique [24]. In brief, the internal aqueous phase (200 μl of 0.1 M HCl) containing 15 mg insulin was added to the organic phase (300 mg polymer dissolved in 2 ml methylene chloride) under vortex mixing for 30 seconds and sonication over 40 seconds in an ice bath using a typed ultrasound nozzle. 4 ml of a 1% aqueous PVA solution were added to the mixture as external aqueous phase under vortex mixing for 10 seconds. The dispersion was then rapidly added into 100 ml of 0.5 % aqueous PVA solution and stirred for 2 hours to evaporate the solvent. The hardened microspheres were centrifuged, washed two times with double-distilled water, freeze-dried and finally stored at −80 °C.

### Investigation of microsphere loading

About 10 mg freeze-dried microspheres were weighed into a 2 ml micro test tube and dissolved in 600 μl of dichloromethane then 600 μl of 0.1 N HCl solution were added and the two phases were vortex mixed. The dispersion was allowed to settle at room temperature for 10 minutes before the dichloromethane phase was separated from the mixture by centrifugation at 2000 rpm for 3 minutes. The upper aqueous phase containing the extracted peptide was analyzed using micro BCA assay and HPLC analysis.

### Microstructure and particle size distribution of insulin-loaded microspheres

Microspheres morphology was investigated after manufacturing and freeze-drying as well as after the release study by scanning electron microscopy (SEM). To investigate their internal structure, microspheres were torn between two layers of adhesive tape. All samples were sputter-coated with a layer of 1.4 nm gold/palladium prior to SEM analysis.

### Insulin release studies

For the investigation of peptide release from biodegradable polymer microspheres, app. 10 mg microspheres were weighed into 2 ml micro test tube and incubated in 1 ml phosphate buffer saline (PBS), pH 7.4 (stabilized with 0.02 % w/v sodium azide). The samples were eroded under gentle shaking (frequency 10 min^−1^) at 37 °C in a shaking water bath. At predetermined time intervals (0, 1, 2, … days) the tubes were centrifuged, and the release medium was withdrawn from each tube and replaced with fresh buffer (kept at the same temperature). All samples were analyzed in triplicate and stored at −80 °C until further analysis.

### Assessment of insulin integrity

The stability of bovine insulin inside polymer microspheres was determined after 6, 12 and 18 days. Microspheres were harvested from the release medium by centrifugation and insulin was extracted using the method described above. The aqueous phase was investigated immediately using HPLC and HPLC-MS analysis.

### High performance liquid chromatography (HPLC) and High performance liquid chromatography-mass spectroscopy (HPLC-MS) analysis

Insulin samples were investigated by HPLC using a setup that consisted of a degasser (from Knauer, Berlin, Germany), LC-10-AT pump, FCV-10ATVP gradient mixer, SIL-10 ADVP autosampler, SPD-10 AV UV detector and SCL-10AVP controller (all from Schimadzu, Duisburg, Germany). A linear gradient of 15% to 40 % solvent B (90% acetonitrile aqueous solution + 0.1% v/v TFA) in solvent A (10% acetonitrile aqueous solution + 0.1% v/v TFA) over 42 minutes was applied as mobile phase at a flow rate of 1.0 ml/min. 100 μl samples were separated at room temperature using a combination of a C18-reversed phase precolumn (LC318, 4.6 mm x 50 mm) and analytical column (LC318, 4.6 mm x 250 mm). Chromatograms were recorded at 274 nm (UV detector).

For HPLC-MS analysis, samples were analysed using an Agilent 1100 HPLC system with API2-source (capillary temperature: 300 °C, spray voltage: 4kV). A linear gradient of 20–95 % solvent B (acetonitrile + 0.1% v/v TFA) in solvent A (double-distilled water + 0.1% v/v TFA) over 30 min served as a mobile phase at a flow rate of 0.2 ml/min. About 10–20μl of the samples were separated using a C18 reversed phase analytical column. The XCALIBUR^®^ software package was used for data acquisition and analysis.
